# Uniform convergence of basic Fourier–Bessel series on a *q*-linear grid

**DOI:** 10.1007/s11139-018-0070-3

**Published:** 2018-09-20

**Authors:** L. D. Abreu, R. Álvarez-Nodarse, J. L. Cardoso

**Affiliations:** 1 Acoustics Research Institute, Austrian Academy of Sciences, Vienna, Austria; 2 Dep. de Análisis Matemático, Universidad de Sevilla, Sevilla, Spain; 3 Dep. de Matemática, Universidade de Trás-os-Montes e Alto Douro (UTAD), Vila Real, Portugal

**Keywords:** Hahn–Exton *q*-Bessel function, Third Jackson *q*-Bessel function, *q*-Fourier series, Basic Fourier expansions, Uniform convergence, *q*-Linear grid, 42C10, 33D15

## Abstract

We study Fourier–Bessel series on a *q*-linear grid, defined as expansions in complete *q*-orthogonal systems constructed with the third Jackson *q*-Bessel function, and obtain sufficient conditions for uniform convergence. The convergence results are illustrated with specific examples of expansions in *q*-Fourier–Bessel series.

## Introduction

Based on the orthogonality relation

$$\begin{aligned} \int _{0}^{1}J_{\nu }(j_{\nu m}t)J_{\nu }(j_{\nu n}t)\mathrm{d}t=0, \end{aligned}$$

if $m\ne n$, where $J_{\nu }$ stands for the Bessel functions of order $\nu$ and $j_{\nu n}$ is their *n*th positive zero, a theory of Fourier–Bessel series was developed (see e.g. [48, §XVIII]), in a close parallelism to the classical theory of Fourier series. Hardy [33] proved that, within some boundaries, the Bessel functions are the most general functions satisfying such an orthogonality “with respect to their own zeros”, giving no space for generalizations of the theory of Fourier–Bessel series in the scope of Lebesgue measure.

However, for a certain *q*-analogue of the Bessel function, such an extension is possible, when considering the proper measure. In the following we will use the standard notation for the *q*-calculus (more precisely, we will follow [31] for basic hypergeometric series, [14] for *q*-calculus and [39] for *q*-Bessel functions). The third Jackson *q*-Bessel function or, for some authors, the Hahn–Exton *q*-Bessel function, is defined as

$$\begin{aligned} J_{\nu }^{(3)}(z;q)\equiv J_{\nu }(z;q):=z^{\nu }\frac{(q^{\nu +1};q)_{\infty }}{(q;q)_{\infty }}\sum \limits _{k=0}^{\infty }(-1)^{k}\frac{ q^{\frac{k(k+1)}{2}}}{(q^{\nu +1};q)_{k}(q;q)_{k}}z^{2k}\text {,} \end{aligned}$$

where $\nu >-1$ is a real parameter. The notation $J_{\nu }^{(k)},k=1,2,3$, from [35] is used to distinguish the three *q*-analogues of the Bessel function defined by Jackson. We will often drop the superscript for notational convenience and simply write $J_{\nu }^{(3)}(z;q)\equiv J_{\nu }(z;q)$. When $q\rightarrow 1^{-}$ one recovers the Bessel function after proper normalization. It is a well known fact [30, 42] that $J_{\nu }(z;q)$ satisfies the orthogonality relation

1.1 $$\begin{aligned}&\int _{0}^{1}xJ_{\nu }(j_{n\nu }qx;q^{2})J_{\nu }(j_{m\nu }qx;q^{2})d_{q}x=\eta _{n,\nu }\delta _{n,m}\text {,}\nonumber \\&\qquad \eta _{n,\nu }= \frac{q-1}{2}q^{\nu -1}J_{\nu +1}(qj_{n\nu };q^{2})J_{\nu }^{\prime }(j_{n\nu };q^{2})\text {,} \end{aligned}$$

where $j_{n\nu }(q^{2})\equiv j_{n\nu }$ are the positive zeros of $J_{\nu }(z;q^{2})$ arranged in ascending order of magnitude, $j_{1\nu }<j_{2\nu }<j_{3\nu }<\cdots$, and $d_{q}x$ stands for the measure of the Jackson *q*-integral.

In the papers [22–24], a theory of Fourier series on a *q*-linear grid was developed, using a *q*-analogue of the exponential function and the corresponding *q*-trigonometric functions introduced by Exton [30]. This was motivated by Bustoz–Suslov orthogonality and completeness results of *q*-quadratic Fourier series [21]. Later, a simple argument has been found to prove such orthogonality and completeness results [37], leading, together with the solution of [38] developed in [7, 8] , to a very general theory of expansions relying on expansion formulas of the plane wave type. The plane wave expansion in Gegenbauer polynomials was extended to the *q*-quadratic case [40] and, more recently, to the *q*-linear case [9] (combined with the orthogonality in [43, Theorem 4.2] this provides an alternative way to derive the results in [1, 5, 42]), using a general method to derive plane-wave type formulas [8]. Since several special function identities can be obtained from Fourier expansions and the associated sampling theorems (see the section by Butzer and Hauss in [34], last section of [1] and the expansions in the last section of this paper for some examples), this may be seen, modulo some poetic licence, as a small contribution towards Gian-Carlo Rota’s 5th problem [46] of finding a unified approach that will give the identities satisfied by both hypergeometric and *q*-hypergeometric functions.

This paper investigates the most delicate convergence issues of the basic Fourier–Bessel series on a *q*-linear grid, based on the orthogonality relation (1.1), on mean convergence results [5, 6], and on the localization of the zeros $j_{n\nu }$ [4]. We will first prove that pointwise convergence associated with orthogonal discrete systems follows directly from the mean convergence. Our main contribution will be a result providing sufficient conditions for uniform convergence. Since it was proved [2], under the same general conditions imposed by Hardy, that the above orthogonality relation characterizes the functions $J_{\nu }^{(3)}(z;q^{2})$, this is the most general Fourier theory based on functions *q*-orthogonal with respect to their own zeros. Moreover, the third Jackson *q*-Bessel function provides a *q*-analogue of the Hankel transform with an inversion formula [44], leading to a full theory of expansions parallel to Fourier theory, including sampling and Paley–Wiener type theorems [1, 3]. As a further evidence of its remarkable structure, we note in passing that the function $J_{\nu }^{(3)}(z;q^{2})$ also shows up naturally in the study of the quantum group of plane motions [41].

It should be emphasized that Ismail stimulated a considerable research activity by conjecturing properties of the zeros of *q*-Bessel functions, confirmed in [4, 32]. First, as documented in [20], the asymptotic expansion for the zeros of *q*-difference equations has been conjectured in a letter from Ismail to Hayman. Then, in a preprint that circulated in the early 2000’s [36], Ismail conjectured properties of the positive zeros of *q*-Bessel functions. Several results followed, among which we can single out [20, 32], the bounds for the zeros of the third Jackson *q*-Bessel function [4], the asymptotic results of [13] and the recent improvement in the corresponding accuracy [47, Prop. A.3]. Since *q*-series provide a wealth of examples of nontrivial functions of order zero, all these results are contributions to the intriguing and relatively overlooked topic of functions of order zero started in Littlewood’s PhD thesis, published in [45]. Since it is well known, by a result of Boas [19, Theorem 5.1], that functions of order zero cannot belong to $$L^{p}(\mathbb {R})$$$$(p\ge 1)$$ without vanishing identically, there is no possibility of expanding them using classical Fourier analysis. Actually it is more a rule than an exception that the methods and concepts used to study functions of positive order do not suffice to study functions of order zero (this is particular notorious if the notion of type is used, which explains why classical tools succeed in [2, 6] and in the study of radii of starlikeness of functions of order zero [10, 17]). Such obstructions lead to the search of alternative methods and provide strong motivation for a theory of expansions in *q*-analogues of classical orthogonal basis functions. In this paper we will investigate series expansions $$S_{q}^{\nu }[f]$$ of functions $$f\in L_{q}^{2}[0,1]$$ of the form

1.2 $$\begin{aligned} S_{q}^{\nu }[f](x)=\sum _{k=1}^{\infty }a_{k}^{\nu }\left( f\right) x^{\frac{1 }{2}}J_{\nu }(qj_{k\nu }x;q^{2})\text {.} \end{aligned}$$

Since the measure of $L_{q}^{2}[0,1]$ is discrete, pointwise convergence is a direct consequence of the completeness results of [5, 6]. We will make some comments about this in Sect. 5 of the paper. In the following we will use the notation $V_{q}^{+}=\left\{ q^{n}:\,n=0,1,2,\ldots \right\}$ for the support points of the *q*-integral (2.3) in [0, 1], and introduce the concept of a *q*-linear Hölder function [24, p. 103].

### Definition 1.1

If two constants *M* and $\lambda$ exist such that

$$\begin{aligned} \Big |f\big (q^{n-1}\big )-f\big (q^{n}\big )\Big |\le Mq^{\lambda n},\quad n=0,1,2,\ldots , \end{aligned}$$

then the function *f* is said to be *q*-linear Hölder of order $\lambda$ (in $V_{q}^{+}\cup \{q^{-1}\}$). If the inequality only holds for $n=1,2,3,\ldots$ then we say that *f* is *q*-linear Hölder of order $\lambda$ in $V_{q}^{+}.$

Our main result is the following sufficient conditions for uniform convergence of (1.2).

### Theorem 1.2

If the function *f* is *q*-linear Hölder of order $\lambda >1$ in $V_{q}^{+}$, such that $t^{-\frac{3}{2}}f(t)\in L_{q}^{2}[0,1]$ and the limit $\lim _{x\rightarrow 0^{+}}f(x)=f(0^{+})$ is finite, the correspondent basic Fourier–Bessel series $S_{q}^{(\nu )}[f](x)$ converges uniformly to *f* on $V_{q}^{+}$ whenever $\nu >0$.

The following is a brief outline of the paper. In the next section, we collect the main definitions and preliminary results. The third section is devoted to the evaluation of a few finite sums. The fourth section contains a brief introduction to *q*-Fourier–Bessel series and the fifth section discusses pointwise convergence for systems associated with discrete orthogonality relations. We prove our main result in Sect. 6, relying on fine estimates for the coefficients of basic Fourier–Bessel series. In the last section of the paper, two examples of basic Fourier–Bessel expansions are provided.

## Definitions and preliminary results

Following the standard notations of [31], consider $0<q<1$, the *q*-shifted factorial for a finite positive integer *n* is defined as

$$\begin{aligned} (a;q)_{n}=\left( 1-q\right) \left( 1-aq\right) \cdots \left( 1-aq^{n-1}\right) ,\qquad (a;q)_{-n}=\frac{1}{\big (aq^{-n};q\big )_{n}} \end{aligned}$$

and the zero and infinite cases as

$$\begin{aligned} (a;q)_{0}=1,\qquad (a;q)_{\infty }=\lim \limits _{n\rightarrow \infty }(a;q)_{n}\text {.} \end{aligned}$$

The symmetric *q*-difference operator acting on a suitable function *f* is defined by

2.1 $$\begin{aligned} \delta _{q}f(x)=f(q^{1/2}x)-f(q^{-1/2}x), \end{aligned}$$

hence, the symmetric *q*-derivative becomes2.2The *q*-integral in the interval $\left( a,b\right)$ is defined by

2.3 $$\begin{aligned} \int _{a}^{b}f\left( t\right) d_{q}t=\int _{0}^{b}f\left( t\right) d_{q}t-\int _{0}^{a}f\left( t\right) d_{q}t \end{aligned}$$

where

$$\begin{aligned} \int _{0}^{a}f\left( t\right) d_{q}t=\left( 1-q\right) \sum _{k=0}^{\infty }f\left( aq^{k}\right) aq^{k}. \end{aligned}$$

This is a Riemann–Stieltjes integral with respect to a step function having infinitely many points of increase at the points $q^{k}$, with the jump at the point $q^{k}$ being *q*. If we call this step function $\mu _{q}(t)$, then $d\mu _{q}(t)=d_{q}t$. One can define an inner product by setting

$$\begin{aligned} \langle f,g\rangle =\int _{0}^{1}f\left( t\right) \overline{g(t)}d_{q}t. \end{aligned}$$

The resulting Hilbert space is commonly denoted by $L_{q}^{2}(0,1)$. The space $L_{q}^{2}(0,1)$ is a separable Hilbert space [11]. For the properties of the more general spaces $L_{q}^{p}(a,b)$ and $L_{q,\omega }^{p}(a,b)$, with $p\ge 1$, see [27]. We will also need the following formula of *q*-integration by parts [25, Lemma 2, p. 327], valid for $a,b\in \mathbb {R}$, assuming the involved limits exist:

2.4 $$\begin{aligned} \int _{a}^{b}g\big (q^{\pm \frac{1}{2}}x\big )\frac{\delta _{q}f(x) }{\delta _{q}x}d_{q}x:= & {} -\int _{a}^{b}f\big (q^{\mp \frac{1}{2}}x \big )\frac{\delta _{q}g(x)}{\delta _{q}x}d_{q}x \nonumber \\&+\, q^{\frac{1}{2}}\left\{ \left[ (fg)(bq^{-\frac{1}{2} })-(fg)(aq^{-\frac{1}{2}})\right] \right. \nonumber \\&\left. -\left[ \lim _{n\rightarrow +\infty }(fg)(bq^{\frac{1}{2}+n}) -\lim _{n\rightarrow +\infty }(fg)(aq^{ \frac{1}{2}+n})\right] \right\} .\nonumber \\ \end{aligned}$$

The third Jackson *q*-Bessel function has a countable infinite number of real and simple zeros [42]. In [4, Theorem 2.3] it was proved that, when $q^{2\nu +2}<(1-q^{2})^{2}$, the positive zeros $j_{k\nu }$ of the function $J_{\nu }(z;q^{2})$ satisfy

2.5 $$\begin{aligned} j_{k\nu }=q^{-k+\epsilon _{k}^{(\nu )}(q^{2})} \end{aligned}$$

with

2.6 $$\begin{aligned} 0<\epsilon _{k}^{(\nu )}(q^{2})<\alpha _{k}^{(\nu )}(q^{2})\text {,} \end{aligned}$$

where

2.7 $$\begin{aligned} \alpha _{k}^{(\nu )}(q^{2})=\frac{\log {\left( 1-q^{2(k+\nu )}/(1-q^{2k})\right) }}{2\log q}\text {.} \end{aligned}$$

Using Taylor expansion it is plain that, as $k\rightarrow \infty$,

2.8 $$\begin{aligned} \alpha _{k}^{(\nu )}(q^{2})=\mathcal {O}\Big (q^{2k}\Big )\text {.} \end{aligned}$$

The restriction $q^{2\nu +2}<(1-q^{2})^{2}$ can be dropped if *k* is chosen large enough [4, Remark 2.5, p. 4247] because (2.5)–(2.7) remain valid for every $k\ge k_{0}$ whenever $q^{2(k_{0}+\nu )}\le \big (1-q^{2}\big )\big (1-q^{2k_{0}} \big )$ . Hence, the following theorem holds.

### Theorem A

For every $q\!\in \,]0,1[,\,k_{0}\in N$ exists such that, if $k\ge k_{0}$ then

$$\begin{aligned} j_{k\nu }=q^{-k+\epsilon _{k}^{(\nu )}(q^{2})}, \end{aligned}$$

where $0<\epsilon _{k}^{(\nu )}(q^{2})<\alpha _{k}^{(\nu )}(q^{2})$ and $\alpha _{k}^{(\nu )}(q^{2})$ is given by (2.7).

In the remaining of the paper we will simplify the notation by setting $\varepsilon _{k}^{(\nu )}=\varepsilon _{k}^{(\nu )}\big (q^{2}\big )$.

The definition of basic Fourier–Bessel series on a *q*-linear grid depends on the following mean convergence result [5, 6].

### Theorem B

The orthonormal sequence $\{u_{k}\}_{k\ge 1}$ defined by $u_{k}^{(\nu )}(x)=\dfrac{x^{\frac{1}{2}}J_{\nu }(j_{k\nu }qx;q^{2})}{ \left\| x^{\frac{1}{2}}J_{\nu }(j_{k\nu }qx;q^{2})\right\| }$ is complete in $L_{q}^{2}(0,1)$.

More precisely, whenever a function *f* is in $L_{q}^{2}(0,1)$ and $\int _{0}^{1}f(x)u_k^{(\nu )}(x)d_{q}x=0, k=1,2,3,\ldots ,$ then $f\big ( q^{k}\big )=0,k=0,1,2,\ldots$. Thus, the orthogonal complement of the space generated by $\{u_k^{(\nu )}\}_{k\ge 1}$ in $L_{q}^{2}(0,1)$ is $\{0\}$ and any $f\in L_{q}^{2}(0,1)$ can be expanded in terms of the sequence $u_{k}^{(\nu )}(x)$.

The following estimate [26] will also be key in the proof of the main results.

### Theorem C

For large values of *k*, $\left| J_{\nu }\big (qj_{k\nu };q^2\big )\right| \le \frac{ \left( -q^2,-q^{2(\nu +1)};q^2\right) _{\infty }}{\left( q^2;q^2\right) _{\infty }} q^{(k+\nu )(k-1)}$.

## Identities for finite sums in *q*-calculus

In this section we gather some new and old identities for finite sums. First observe that one can rewrite the obvious identity

$$\begin{aligned} (a;q)_{m}\big (aq^{m};q\big )_{k}=(a;q)_{k}\big (aq^{k};q\big )_{m}, \end{aligned}$$

which holds for every *a* and for every integers *m* and *k*, as

3.1 $$\begin{aligned} \frac{\big (aq^{m};q\big )_{k}}{(a;q)_{k}}=\frac{\big (aq^{k};q\big )_{m}}{ (a;q)_{m}} \end{aligned}$$

valid for any $a\ne q^{-j}$, $j=0,1,2,\ldots ,$ and *m* and *k* nonnegative integers.

### Proposition 3.1

For each complex *a*, the identity

$$\begin{aligned} \sum _{k=0}^{n}q^{k}\frac{\big (a;q\big )_{k}}{(q;q)_{k}}=\frac{\big (aq;q\big ) _{n}}{(q;q)_{n}} \end{aligned}$$

holds for all $n=0,1,2,\ldots .$

### Proof

We argue by induction. The proposition is obvious when $n=0$. For the induction step, one can write the sum for $n+1$ as

$$\begin{aligned} \sum _{k=0}^{n+1}q^{k}\frac{\big (a;q\big )_{k}}{(q;q)_{k}}&=\sum _{k=0}^{n}q^{k} \frac{\big (a;q\big )_{k}}{(q;q)_{k}}+q^{n+1}\frac{\big (a;q\big )_{n+1}}{ (q;q)_{n+1}}\\&=\frac{\big (aq;q\big )_{n}}{(q;q)_{n}}+q^{n+1}\frac{\big (a;q\big ) _{n+1}}{(q;q)_{n+1}}\text {.} \end{aligned}$$

From the induction hypothesis,

$$\begin{aligned} \sum _{k=0}^{n+1}q^{k}\frac{\big (a;q\big )_{k}}{(q;q)_{k}}&=\frac{\big (aq;q\big ) _{n}}{(q;q)_{n}}\left( 1+q^{n+1}\frac{1-a}{1-q^{n+1}}\right) \\&=\frac{\big (aq;q \big )_{n}}{(q;q)_{n}}\frac{1-aq^{n+1}}{1-q^{n+1}}=\frac{\big (aq;q\big )_{n+1} }{(q;q)_{n+1}}. \end{aligned}$$

$$\square $$

### Proposition 3.2

For each *a* and $\lambda$ in the complex plane, the identity

$$\begin{aligned} \sum _{k=0}^{n}q^{2k}\frac{\big (\frac{a}{q};q\big )_{k}}{(q;q)_{k}}\Big ( q^{1+n-k+\lambda };q\Big )_{1}=(q;q)_{1}\frac{(aq;q)_{n}}{(q;q)_{n}}+\big ( q^{\lambda };q\big )_{1}q^{1+n}\frac{(a;q)_{n}}{(q;q)_{n}} \end{aligned}$$

holds for all $n=0,1,2,\ldots .$

### Proof

We will perform the proof in two steps. First, using induction on *n*, we prove the case $\lambda =0$:

3.2 $$\begin{aligned} \sum _{k=0}^{n}q^{2k}\frac{\big (\frac{a}{q};q\big )_{k}}{(q;q)_{k}}\Big ( q^{1+n-k};q\Big )_{1}=(q;q)_{1}\frac{(aq;q)_{n}}{(q;q)_{n}} \end{aligned}$$

and then reduce the general case to this particular one.

(i) *Step 1*: $\lambda =0$. For $n=0$, the identity is trivial. For the induction step one decomposes the sum as

$$\begin{aligned}&\sum _{k=0}^{n}q^{2k}\frac{\big (\frac{a}{q};q\big )_{k}}{(q;q)_{k}}\Big ( q^{1+n-k};q\Big )_{1} \\&\quad =\sum _{k=0}^{n-1}q^{2k}\frac{\big (\frac{a}{q};q\big )_{k}}{(q;q)_{k}}\Big ( q^{1+n-k};q\Big )_{1}+q^{2n}\frac{\big (\frac{a}{q};q\big )_{n}}{(q;q)_{n}} (q;q)_{1} \\&\quad =\sum _{k=0}^{n-1}q^{2k}\frac{\big (\frac{a}{q};q\big )_{k}}{(q;q)_{k}}\big ( q^{n-k};q)_{1}+(q;q)_{1}q^{n}\sum _{k=0}^{n-1}q^{k}\frac{\big (\frac{a}{q};q \big )_{k}}{(q;q)_{k}}+q^{2n}\frac{\big (\frac{a}{q};q\big )_{n}}{(q;q)_{n}} (q;q)_{1}\text {,} \end{aligned}$$

using $\big (q^{1+n-k};q\big )_{1}=1-q^{1+n-k}=1-q^{n-k}+q^{n-k}(1-q)=\big ( q^{n-k};q)_{1}+q^{n-k}(q;q)_{1}$ in the last identity. Combining the induction hypothesis with Proposition 3.1 yields

$$\begin{aligned} \sum _{k=0}^{n}q^{2k}\frac{\big (\frac{a}{q};q\big )_{k}}{(q;q)_{k}}\Big ( q^{1+n-k};q\Big )_{1}&=(q;q)_{1}\frac{(aq;q)_{n-1}}{(q;q)_{n-1}} +(q;q)_{1}q^{n}\frac{(a;q)_{n-1}}{(q;q)_{n-1}}\\&\qquad + \, q^{2n}\frac{\big (\frac{a}{q};q \big )_{n}}{(q;q)_{n}}(q;q)_{1} \\&=\frac{\big (aq;q\big )_{n-2}}{(q;q)_{n}}(q;q)_{1}\left( 1-aq^{n-1}\right) \left( 1-aq^{n}\right) \\&=\frac{\big (aq;q\big )_{n}}{(q;q)_{n}}(q;q)_{1}\text {,} \end{aligned}$$

leading to (3.2).

(ii) *Step 2*: $\lambda \in \mathbb {C}$. First notice that

$$\begin{aligned} \big (q^{1+n-k+\lambda };q\big )_{1}=1-q^{1+n-k+\lambda }=1-q^{1+n-k}+q^{1+n-k} \big (1-q^{\lambda }\big ) \end{aligned}$$

and then use Proposition 3.1 and the case $\lambda =0$ (3.2) of the first step. $\square$

### Lemma 3.3

For each complex *a* and each non-negative integer *i*, the identity

$$\begin{aligned} \sum _{k=0}^{i}q^{2k}\frac{\big (\frac{a}{q};q\big )_k}{(q;q)_k}\left( \sum _{j=0}^{n}q^{j}\frac{\big (aq^{i};q\big )_{j}}{\big (q^{1+i};q\big )_{j}} \frac{\big (q^{1+i+j-k};q\big )_1}{(q^{1+i+j};q)_1}\right) = \frac{(aq;q)_{n+i} }{(q;q)_{n+i}}\frac{\big (q^{1+n};q\big )_1}{\big (q^{1+n+i};q\big )_1} \end{aligned}$$

holds for all $n=0,1,2,\ldots .$

### Proof

We use induction over *n* . The case $n=0$ is precisely identity (3.2) and writing

$$\begin{aligned}&\sum _{k=0}^{i}q^{2k}\frac{\big (\frac{a}{q};q\big )_{k}}{(q;q)_{k} }\left( \sum _{j=0}^{n+1}q^{j}\frac{\big (aq^{i};q\big )_{j}}{\big (q^{1+i};q \big )_{j}}\frac{\big (q^{1+i+j-k};q\big )_{1}}{(q^{1+i+j};q)_{1}}\right) \\&\quad =\sum _{k=0}^{i}q^{2k}\frac{\big (\frac{a}{q};q\big ) _{k}}{(q;q)_{k}}\left( \sum _{j=0}^{n}q^{j}\frac{\big (aq^{i};q\big )_{j}}{\big ( q^{1+i};q\big )_{j}}\frac{\big (q^{1+i+j-k};q\big )_{1}}{(q^{1+i+j};q)_{1}} \right) \\&\qquad + \, q^{n+1}\frac{\big (aq^{i};q\big )_{n+1}}{\big (q^{1+i};q\big )_{n+2}} \sum _{k=0}^{i}q^{2k}\frac{\big (\frac{a}{q};q\big )_{k}}{(q;q)_{k}}\big ( q^{n+2+i-k};q\big )_{1} \end{aligned}$$

the result follows from algebraic manipulations, after combining the induction hypothesis with Proposition 3.2. $\square$

### Remark 3.4

Using identity (3.1) Proposition 3.1 can be written as

$$\begin{aligned} \sum _{k=0}^{i}q^{k}\frac{\big (q^{1+k};q\big )_{j-1}}{(q;q)_{j-1}}=\frac{\big ( q^{1+i};q\big )_{j}}{(q;q)_{j}}\text {.} \end{aligned}$$

Now we will consider the sum

$$\begin{aligned} a_{0}^{(n,\nu )}:=q^{-n\nu }\sum _{i=0}^{n}\big (q^{2\nu }\big )^{i}\text {,} \end{aligned}$$

where *n* is a nonnegative integer and $\nu$ is a fixed parameter, which will show up in Sect. 6. The notation [*x*] to denote the greatest integer which does not exceed *x* will be adopted.

### Lemma 3.5

For a given sequence $\left\{ \gamma _{\lambda }\right\}$ we have, for $m=0,1,2,\ldots$,

$$\begin{aligned} \sum _{\lambda =0}^{m}a_{0}^{(\lambda ,\nu )}a_{0}^{(m-\lambda ,\nu )}\gamma _{\lambda }=\sum _{\theta =0}^{\left[ \frac{m}{2}\right] }a_{0}^{(m-2\theta ,\nu )}\left( \sum _{\lambda =\theta }^{m-\theta }\gamma _{\lambda }\right) \text {.} \end{aligned}$$

### Proof

Using induction on *m* it can be proved that

3.3 $$\begin{aligned} a_{0}^{(\lambda ,\nu )}a_{0}^{(m-\lambda ,\nu )}=\sum _{\theta =0}^{\lambda }a_{0}^{(m-2\theta ,\nu )}\quad \hbox {if}\qquad 0\le \lambda \le \left[ \frac{m}{2}\right] \end{aligned}$$

holds for all $m=0,1,2,\ldots$ . As a consequence,

3.4 $$\begin{aligned} a_{0}^{(\lambda ,\nu )}a_{0}^{(m-\lambda ,\nu )}=\sum _{\theta =0}^{m-\lambda }a_{0}^{(m-2\theta ,\nu )}\quad \hbox {if}\qquad \left[ \frac{m}{2}\right] +1\le \lambda \le m\text {.} \end{aligned}$$

Writing

$$\begin{aligned} \sum _{\lambda =0}^{m}a_{0}^{(\lambda ,\nu )}a_{0}^{(m-\lambda ,\nu )}\gamma _{\lambda }=\sum _{\lambda =0}^{\left[ \frac{m}{2}\right] }a_{0}^{(\lambda ,\nu )}a_{0}^{(m-\lambda ,\nu )}\gamma _{\lambda }+\sum _{\lambda =\left[ \frac{m}{2}\right] +1}^{m}a_{0}^{(\lambda ,\nu )}a_{0}^{(m-\lambda ,\nu )}\gamma _{\lambda }\text { ,} \end{aligned}$$

the lemma follows from (3.3), (3.4) and some algebra. $\square$

## Fourier–Bessel series on a *q*-linear grid

The Fourier–Bessel series on a *q*-linear grid associated with *f* is defined as the sum

$$\begin{aligned} S_{q}^{\nu }[f](x)&=\sum _{k=1}^{\infty }a_{k}^{\nu }\left( f\right) x^{\frac{1 }{2}}J_{\nu }(qj_{k\nu }x;q^{2}),\\&\quad \hbox {being}\quad a_{k}^{\nu }\left( f\right) =\frac{1}{\eta _{k,\nu }}\int _{0}^{1}t^{\frac{1}{2}}f(t)J_{\nu }(qj_{k\nu }t;q^{2})d_{q}t \end{aligned}$$

or, equivalently,

4.1 $$\begin{aligned} S_{q}^{(\nu )}[f](x)=\sum _{k=1}^{\infty }a_{k}^{(\nu )}\left( f\right) J_{\nu }(qj_{k\nu }x;q^{2}), \end{aligned}$$

with the coefficients $a_{k}^{(\nu )}$ given as

4.2 $$\begin{aligned} a_{k}^{(\nu )}\left( f\right) =\frac{1}{\eta _{k,\nu }}\int _{0}^{1}tf(t)J_{ \nu }(qj_{k\nu }t;q^{2})d_{q}t, \end{aligned}$$

and

4.3 $$\begin{aligned} \eta _{k,\nu }=\int _{0}^{1}\left[ t^{\frac{1}{2}}J_{\nu }\big ( qj_{k\nu }t;q^{2}\big )\right] ^{2}d_{q}t= & {} -\dfrac{1-q}{2}q^{\nu -1}J_{\nu +1}(qj_{k\nu };q^{2})J_{\nu }^{\prime }(j_{k\nu };q^{2})\nonumber \\= & {} -\frac{(1-q)q^{\nu -2}}{2j_{k\nu }}J_{\nu }(qj_{k\nu };q^{2})J_{\nu }^{\prime }(j_{k\nu };q^{2}).\nonumber \\ \end{aligned}$$

The last equality in formula (4.3) follows from the identity (see, e.g., [25, Prop. 5 (vii), p. 330])

4.4 $$\begin{aligned} J_{\nu }\big (qj_{k\nu };q^{2}\big )=qj_{k\nu }J_{\nu +1}\big (qj_{k\nu };q^{2} \big ). \end{aligned}$$

Theorem B assures mean convergence of the series (4.1). In the next two sections we will see that it also converges at each of the points $x\in V_{q}^{+}=\left\{ q^{n}:n=0,1,2,\ldots \right\}$ and obtain sufficient conditions for its uniform convergence.

## Pointwise convergence

### A general set-up

With a view to studying pointwise convergence of the series (4.1) when $x\in V_{q}^{+}=\left\{ q^{n}:n=0,1,2,\ldots \right\}$, we first establish a general result in a more general setting, designed to cover not only the convergence of *q*-Fourier–Bessel series but also other Fourier systems based on discrete orthogonality relations, as in [12, 22–24]. There is no real novelty in this section and we are aware that the pointwise convergence follows from the mean convergence by using known results from linear analysis. However, we believe that the reader may benefit from the following elegant self contained argument, which has been gently provided to us by Professor Juan Arias de Reyna.

Let $\mathcal {N}=\{a_{n}|\,n\in {\mathbb {N}}\}$ be a numerable space and let $\mu$ be a positive measure on $\mathcal {N}$ such that $\mu _{n}=\mu (\{a_{n}\})>0$. We will denote by $\mathcal {L}_{\mu }^{2}$, the space of all functions $f:\mathcal {N}\mapsto {\mathbb {C}}$, such that

$$\begin{aligned} \Vert f\Vert _{\mathcal {L}_{\mu }^{2}}^{2}=\sum _{n=1}^{\infty }|f(a_{n})|^{2}\mu _{n}<+\infty . \end{aligned}$$

In such a space, the scalar product $\langle f,g\rangle$ of two functions is defined by

$$\begin{aligned} \langle f,g\rangle _{\mu }=\sum _{n=1}^{\infty }f(a_{n})\overline{g(a_{n})} \mu _{n}. \end{aligned}$$

The sequence of functions $(e_{n})_{n\ge 1}$ defined on $\mathcal {N}$ by

5.1 $$\begin{aligned} e_{n}(a_{k})=\left\{ \begin{array}{ll} \mu _{n}^{-1/2}, &{}\quad k=n, \\ 0, &{}\quad k\ne n. \end{array} \right. \end{aligned}$$

is a complete orthonormal system in $\mathcal {L}_{\mu }^{2}$. To check this fact, notice that the function $g_{N}$, $N\in {\mathbb {N}}$, defined by

$$\begin{aligned} g_{N}=f-\sum _{n=1}^{N}\langle f,e_{n}\rangle _{\mu }e_{n},\qquad f\in \mathcal {L}_{\mu }^{2}, \end{aligned}$$

is such that $g_{N}(a_{k})=0$ for all $k\le N$ and $g_{N}(a_{k})=f(a_{k})$ for all $k>N$. Therefore,

$$\begin{aligned} \Vert g_{N}\Vert _{\mathcal {L}_{\mu }^{2}}^{2}=\sum _{n=N+1}^{\infty }|f(a_{n})|^{2}\mu _{n}\rightarrow 0,\,\hbox {as}\,N\rightarrow \infty . \end{aligned}$$

Thus, for an arbitrary $f\in \mathcal {L}_{\mu }^{2}$, we have

$$\begin{aligned} f=\sum _{n=1}^{\infty }\langle f,e_{n}\rangle _{\mu }e_{n}, \end{aligned}$$

with convergence in norm $\Vert \cdot \Vert _{\mathcal {L}_{\mu }^{2}}^{2}$ . This is also true for any other complete orthonormal system $(u_{n})_{n\ge 1}$, i.e., for an arbitrary $f\in \mathcal {L}_{\mu }^{2}$ one has

$$\begin{aligned} f=\sum _{n=1}^{\infty }\langle f,u_{n}\rangle _{\mu }u_{n}, \end{aligned}$$

with convergence in norm $\Vert \cdot \Vert _{\mathcal {L}_{\mu }^{2}}^{2}$. In the following lemma it is shown that this convergence also holds pointwise.

#### Lemma 5.1

Let $(u_n)_{n\ge 1}$ be a complete orthonormal system in $\mathcal {L}^2_{\mu }$. Then for any arbitrary $f\in \mathcal {L}^2_{\mu }$

$$\begin{aligned} f(a_k)=\lim _{N\rightarrow \infty }\sum _{n=1}^N \langle f, u_n\rangle u_n(a_k), \quad \forall \,a_k\in \mathcal {N}. \end{aligned}$$

#### Proof

Let $a_k$ be an arbitrary element of $\mathcal {N}$. Then, the function $d_k:=\mu _k^{-1/2}e_k$, where $e_k$ is the function given in (5.1), satisfies the property

$$\begin{aligned} \langle f, d_k\rangle =\langle f, \mu _k^{-1/2}e_k\rangle =f(a_k)\mu _k^{-1/2} e_k(a_k)\mu _k=f(a_k). \end{aligned}$$

In particular, $\langle u_n, d_k \rangle =u_n(a_k)$. Then,

$$\begin{aligned} f(a_k)=\langle f, d_k\rangle =\left\langle \lim _{N\rightarrow \infty }\sum _{n=1}^N\langle f, u_n\rangle u_n, d_k \right\rangle = \lim _{N\rightarrow \infty } \sum _{n=1}^N\langle f, u_n\rangle \langle u_n, d_k \rangle , \end{aligned}$$

and, therefore,

$$\begin{aligned} f(a_k)=\lim _{N\rightarrow \infty } \sum _{n=1}^N \langle f, u_n\rangle u_n(a_k). \end{aligned}$$

$$\square $$

### Application to *q*-Fourier–Bessel series

Let $\mathcal {N}=V_{q}^{+}$ and $\mu$ the measure associated to the Jackson *q*-integral (2.3). The corresponding $\mathcal {L}_{\mu }^{2}$ space, denoted by $L_{q}^{2}[0,1]$ is equipped with the norm

$$\begin{aligned} \left( \int _{0}^{1}|f(x)|^{2}d_{q}x\right) ^{\frac{1}{2}}<+\infty , \end{aligned}$$

Since the set of functions

$$\begin{aligned} u_{n}^{(\nu )}(x)=\frac{x^{\frac{1}{2}}J_{\nu }(j_{n\nu }qx;q^{2})}{ \left\| x^{\frac{1}{2}}J_{\nu }(j_{n\nu }qx;q^{2})\right\| _{L_{q}^{2}[0,1]}}, \end{aligned}$$

is a complete orthonormal system in $L_{q}^{2}[0,1]$ then, for an arbitrary $f\in L_{q}^{2}[0,1]$, i.e., we have the equality

$$\begin{aligned} f(q^{k})=\lim _{N\rightarrow \infty }\sum _{n=1}^{N}\langle f,u_{n}^{(\nu )}\rangle u_{n}^{(\nu )}(q^{k}),\quad k=0,1,2,\ldots , \end{aligned}$$

where

$$\begin{aligned} \langle f,u_{n}^{(\nu )}\rangle =\int _{0}^{1}f(t)u_{n}^{(\nu )}(t)d_{q}t. \end{aligned}$$

Applying the results of the previous section leads to the following theorem.

#### Theorem 5.2

If $f\in L_{q}^{2}[0,1]$, then the *q*-Fourier–Bessel series (4.1) converges to the function *f* at every point $x\in V_q^{+}$.

#### Remark 5.3

In the case of the standard trigonometric series the equivalent result of Lemma 5.1 ($\mathcal {L}_{\mu }^{2}$ convergence implies pointwise convergence) is not true. In fact this problem leads to the celebrated Carleson Theorem ([28]; for a tutorial exposition see [16]). The main difference between these two cases is that $L_{q}^{2}[0,1]$ is a reproducing kernel Hilbert space, while $L^{2}[0,2\pi ]$ is not. More precisely, in contrast to $\mathcal {L}_{\mu }^{2}$ (see the function $d_{k}$ used in the proof of Lemma 5.1), for functions $f\in L^{2}[0,2\pi ]$ and for every $a\in [0,2\pi ]$, there exists no function $f_{a}$ such that $\langle f,f_{a}\rangle =f(a)$.

#### Remark 5.4

In [23], convergence theorems for *q*-Fourier series associated with the *q*-trigonometric orthogonal system $\Big \{1,\,C_{q}\big (q^{\frac{1}{2} }\omega _{k}x\big ),\,S_{q}\left( q\omega _{k}x\right) \Big \}$ were established, where the *q*-cosines $C_{q}$ and *q*-sinus $S_{q}$ can be defined in terms of the third *q*-Bessel functions by the identities

$$\begin{aligned} C_{q}(z)&=q^{-3/8}\frac{(q^{2};q^{2})_{\infty }}{(q;q^{2})_{\infty }} z^{1/2}J_{-1/2}\big (q^{-3/4}z;q^{2}\big ),\\ S_{q}(z)&=q^{1/8}\frac{ (q^{2};q^{2})_{\infty }}{(q;q^{2})_{\infty }}z^{1/2}J_{1/2}\big ( q^{-1/4}z;q^{2}\big )\;, \end{aligned}$$

where $$\left\{ \omega _{k}\right\} $$ is the sequence of positive zeros of the function $$S_{q}$$, arranged in ascendant order of magnitude. Since this orthogonal system is a complete system (see, e.g., [22]) in $$ L_{q}^{2}[-1,1]$$, the *q*-trigonometric Fourier series defined in [23] converges to $$f\in L_{q}^{2}[-1,1]$$ at every point of $$V_{q}\,=\,\left\{ \pm q^{n}:\,n=0,1,2,\ldots \right\} $$: for every $$x\in V_{q}$$, the identity

$$\begin{aligned} f(x)=\frac{a_{0}}{2}+\sum _{k=1}^{\infty }\left\{ a_{k}C_{q}\big (q^{\frac{1}{2 }}\omega _{k}x\big )+b_{k}S_{q}\big (q\omega _{k}x\big )\right\} , \end{aligned}$$

holds with $a_{0}=\int _{-1}^{1}f(t)d_{q}t$ and

$$\begin{aligned} a_{k}=\frac{1}{\tau _{k}}\int _{-1}^{1}f(t)C_{q}\big (q^{\frac{1}{2}}\omega _{k}t\big )d_{q}t,\quad b_{k}=\frac{q^{\frac{1}{2}}}{\tau _{k}} \int _{-1}^{1}f(t)S_{q}\left( q\omega _{k}t\right) d_{q}t, \end{aligned}$$

for $k=1,2,3,\ldots$, where $\tau _{k}=(1-q)C_{q}(q^{1/2}\omega _{k})S_{q}^{\prime }(\omega _{k})$. This answers a question posed in the concluding section of [23].

#### Remark 5.5

In [12] a rigorous theory of *q*-Sturm–Liouville systems was developed. In particular, it was shown that the set of all normalized eigenfunctions forms an orthonormal basis for $L_{q}^{2}[0,a]$. Therefore Lemma 5.1 can be used to show that the Fourier expansions in terms of the eigenfunctions of *q*-Sturm–Liouville systems are pointwise convergent.

## Uniform convergence

By (4.1) and (4.2) one may write, with $\eta _{k,\nu }$ given by (4.3),

6.1 $$\begin{aligned} S_{q}^{(\nu )}[f](q^{n})= & {} \sum _{k=1}^{\infty } a_{k}^{(\nu )}\left( f\right) J_{\nu }\big (q^{n+1}j_{k\nu };q^{2}\big ) \nonumber \\= & {} \sum _{k=1}^{\infty }\left( \frac{1}{\eta _{k,\nu }} \int _{0}^{1} tf(t)J_{\nu }\big (qj_{k\nu }t;q^{2}\big )d_{q}t\right) J_{\nu } \big (q^{n+1}j_{k\nu };q^{2}\big ). \end{aligned}$$

### Behavior of $J_{\nu }\big (q^{n+1}j_{k\nu };q^{2} \big )$

#### Proposition 6.1

For $$n=0,1,2,\ldots $$,

$$\begin{aligned} J_{\nu }\big (q^{n+1}j_{k\nu };q^{2}\big )=J_{\nu }\big (qj_{k\nu };q^{2}\big ) P_{n}\big (j_{k\nu }^{2};q\big ), \end{aligned}$$

where $$\left\{ P_{n}(x;q)\right\} _{n}$$ is a sequence of polynomials such that, for each $$n=0,1,2,\ldots ,$$$$P_{n}(x;q)$$ has degree *n* in the variable *x* and

$$\begin{aligned} \left\{ \begin{array}{l} P_{n+1}\big (j_{k\nu }^{2};q\big )=\Big \{\big (q^{\nu }+q^{-\nu }\big )-q^{-\nu +2(n+1)}j_{k\nu }^{2}\Big \}P_{n}\big (j_{k\nu }^{2};q\big )-P_{n-1}\big ( j_{k\nu }^{2};q\big ), \\ P_{0}(j_{k\nu }^{2};q)=1,\quad P_{-1}(j_{k\nu }^{2};q)=0. \end{array} \right. \end{aligned}$$

#### Proof

Consider the basic difference relation (2.12) of [42, p. 693]

$$\begin{aligned} J_{\nu }\big (q^{2}x;q^{2}\big )+q^{-\nu }\big (q^{2}x^{2}-1-q^{2\nu }\big ) J_{\nu }\big (qx;q^{2}\big )+J_{\nu }\big (x;q^{2}\big )=0 \end{aligned}$$

Setting $x=q^{n-1}j_{k,\nu }$, the proposition follows using induction on *n*. $\square$

Writing

6.2 $$\begin{aligned} P_{n}\big (j_{k\nu }^{2};q\big ):=\sum _{j=0}^{n}a_{j}^{(n,\nu )}(q)\big ( j_{k\nu }^{2}\big )^{j}\text {,} \end{aligned}$$

Proposition 6.1 leads to the following recurrence relation for the polynomial coefficients $a_{j}^{(n,\nu )}\equiv a_{j}^{(n,\nu )}(q)$:

6.3 $$\begin{aligned} \left\{ \begin{array}{l} a_{j}^{(n+1,\nu )}=\big (q^{\nu }+q^{-\nu }\big )a_{j}^{(n,\nu )}-q^{-\nu +2(n+1)}a_{j-1}^{(n,\nu )}-a_{j}^{(n-1,\nu )}\;\;,\quad j\le n\,; \\ a_{0}^{(0,\nu )}=1\;;\quad a_{-1}^{(i,\nu )}=0\quad \hbox {whenever}\quad i\ge 0\,; \\ a_{j}^{(n,\nu )}=0\quad \hbox {whenever}\quad j>n. \end{array} \right. \end{aligned}$$

Although *n* and *j* are nonnegative integers, relation (6.3) can be considered for any integers *n* and *j*. Moreover, it follows from (6.3) that, for every integer *n*,

6.4 $$\begin{aligned} a_{0}^{(n,\nu )}=q^{-n\nu }\sum _{i=0}^{n}\big (q^{2\nu }\big )^{i}\,\quad \hbox {and}\quad a_{n}^{(n,\nu )}=(-1)^{n}q^{n(n+1-\nu )}\,\text {,} \end{aligned}$$

and

6.5 $$\begin{aligned} a_{0}^{(n,\nu )}=a_{0}^{(1,\nu )}a_{0}^{(n-1,\nu )}-a_{0}^{(n-2,\nu )}. \end{aligned}$$

The first identity of (6.4) can be obtained by iterating (6.3) and the second identity by iterating $a_{n}^{(n,\nu )}=q^{-\nu +2n}a_{n-1}^{(n-1,\nu )}$, which is also a consequence of (6.3). Noticing that $a_{0}^{(1,\nu )}=q^{\nu }+q^{-\nu }$ and replacing *n* by $n-1$, the recurrence relation (6.3) may be further rewritten in the form

$$\begin{aligned} a_{j}^{(n,\nu )}=a_{0}^{(1,\nu )}a_{j}^{(n-1,\nu )}-a_{j}^{(n-2,\nu )}-q^{-\nu +2n}a_{j-1}^{(n-1,\nu )}. \end{aligned}$$

Replacing *n* by $n-2$ in (6.3) and inserting the resulting expression for $a_{j}^{(n-1,\nu )}$ from the previous identity,

$$\begin{aligned} a_{j}^{(n,\nu )}= & {} \left( \big (a_{0}^{(1,\nu )}\big )^{2}-1\right) a_{j}^{(n-2,\nu )}-a_{0}^{(1,\nu )}a_{j}^{(n-3,\nu )}\\&- \, q^{-\nu }\left[ q^{2n}a_{j-1}^{(n-1,\nu )}+q^{2(n-1)}a_{0}^{(1,\nu )}a_{j-1}^{(n-2,\nu )} \right] \\= & {} a_{0}^{(2,\nu )}a_{j}^{(n-2,\nu )}-a_{0}^{(1,\nu )}a_{j}^{(n-3,\nu )}\\&-q^{-\nu }\left[ q^{2n}a_{j-1}^{(n-1,\nu )}+ \, q^{2(n-1)}a_{0}^{(1,\nu )}a_{j-1}^{(n-2,\nu )}\right] , \end{aligned}$$

where (6.5) was used for the last identity.

Repeating the same argument for $a_{j}^{(n-2,\nu )}$ and using (6.5),

$$\begin{aligned} a_{j}^{(n,\nu )}= & {} \left( a_{0}^{(1,\nu )}a_{0}^{(2,\nu )}-a_{0}^{(1,\nu )}\right) a_{j}^{(n-3,\nu )}-a_{0}^{(2,\nu )}a_{j}^{(n-4,\nu )} \\&- \, q^{-\nu }\left[ q^{2n}a_{j-1}^{(n-1,\nu )}+q^{2(n-1)}a_{0}^{(1,\nu )}a_{j-1}^{(n-2,\nu )}+q^{2(n-2)}a_{0}^{(2,\nu )}a_{j-1}^{(n-3,\nu )}\right] \\= & {} a_{0}^{(3,\nu )}a_{j}^{(n-3,\nu )}-a_{0}^{(2,\nu )}a_{j}^{(n-4,\nu )}\\&- \, q^{-\nu }\left[ q^{2n}a_{j-1}^{(n-1,\nu )}+q^{2(n-1)}a_{0}^{(1,\nu )}a_{j-1}^{(n-2,\nu )}+q^{2(n-2)}a_{0}^{(2,\nu )}a_{j-1}^{(n-3,\nu )}\right] . \end{aligned}$$

Iterating $n-j$ times the same argument provides the identity

$$\begin{aligned} a_{j}^{(n,\nu )}=a_{0}^{(n-j,\nu )}a_{j}^{(j,\nu )}-a_{0}^{(n-j-1,\nu )}a_{j}^{(j-1,\nu )}-q^{-\nu }\sum _{\lambda =0}^{n-1-j}q^{2(n-\lambda )}a_{0}^{(\lambda ,\nu )}a_{j-1}^{(n-1-\lambda ,\nu )}. \end{aligned}$$

Since $$a_{j}^{(j-1,\nu )}=0$$ and $$a_{j}^{(j,\nu )}=-q^{-\nu +2j}a_{j-1}^{(j-1,\nu )}$$,

$$\begin{aligned} a_{j}^{(n,\nu )}=-q^{-\nu }\sum _{\lambda =0}^{n-j}q^{2(n-\lambda )}a_{0}^{(\lambda ,\nu )}a_{j-1}^{(n-1-\lambda ,\nu )}, \end{aligned}$$

and, therefore,

6.6 $$\begin{aligned} \left\{ \begin{array}{l} a_{j}^{(n,\nu )}=-q^{2-\nu }\sum _{\lambda =0}^{n-j}q^{2(n-1-\lambda )}a_{0}^{(\lambda ,\nu )}a_{j-1}^{(n-1-\lambda ,\nu )}\;\;,\quad j\le n\,; \\ a_{0}^{(0,\nu )}=1\;;\quad a_{-1}^{(i,\nu )}=0\quad \hbox {whenever}\quad i\ge 0\,; \\ a_{j}^{(n,\nu )}=0\quad \hbox {whenever}\quad j>n. \end{array} \right. \end{aligned}$$

The following result uses (6.6) to compute the quantities $a_{j}^{(n,\nu )}$ in explicit form.

#### Proposition 6.2

An explicit expression for the polynomial coefficients $a_j^{(n,\nu )}$ is given by

$$\begin{aligned} a_{j}^{(n,\nu )}= & {} (- 1)^{j}q^{j(j+1-\nu )} \!\sum _{i=0}^{\left[ \frac{n-j}{2}\right] }\!a_{0}^{(n-j-2i,\nu )}q^{2i} \frac{\left( \left( q^2\right) ^{ j}\!;q^2\right) _{i}}{\left( q^2;q^2\right) _{i}} \frac{ \left( \left( q^2\right) ^{1+j}\!;q^2\right) _{n-j-2i}}{ \left( q^2;q^2\right) _{n-j-2i}}\\&\quad \times \frac{\left( \left( q^2\right) ^{1 +n-2i}\!;q^2\right) _{i}}{ \left( \left( q^2\right) ^{n-j- 2i+2}\!;q^2\right) _{i}} \\= & {} (-1)^{j}q^{j(j+1-\nu )} \!\sum _{i=0}^{\left[ \frac{n-j}{2}\right] }a_{0}^{(n-j-2i,\nu )}q^{2i} \frac{ \left( \left( q^2\right) ^{j}\!;q^2\right) _{i}}{\left( q^2;q^2 \right) _{i}} \frac{\left( \left( q^2\right) ^{1+j}\!;q^2\right) _{ n-j-i}}{\left( q^2;q^2\right) _{n-j-i}}\\&\quad \times \frac{ \left( \left( q^2\right) ^{1+n-j-2i}\!;q^2\right) _{1}}{ \left( \big (q^2\big )^{1+n-j-i}\!;q^2\right) _{1}}, \end{aligned}$$

with $$0\le j\le n$$, $$n=0,1,2,\ldots .$$

#### Proof

The proof, once again carried out by induction, is a bit long and technical. We will simply present a sketch. The case $n=0$ is obvious. We point out that Proposition 6.2 is true for every *n* when $j=0$ . Let us now assume that

$$\begin{aligned} a_{l}^{(k,\nu )}= & {} (-1)^{l}q^{l(l+1-\nu )}\!\sum _{i=0}^{\left[ \frac{ k-l}{2}\right] }a_{0}^{(k-l-2i,\nu )}q^{2i}\frac{\left( \left( q^{2}\right) ^{l}\!;q^{2}\right) _{i}}{\left( q^{2};q^{2}\right) _{i}}\frac{\left( \left( q^{2}\right) ^{1+l}\!;q^{2}\right) _{k-l-2i}}{\left( q^{2};q^{2}\right) _{k-l-2i}}\\&\quad \times \frac{\left( \left( q^{2}\right) ^{1+k-2i}\!;q^{2}\right) _{i}}{\left( \left( q^{2}\right) ^{k-l-2i+2}\!;q^{2}\right) _{i}} \end{aligned}$$

holds true for $k=0,1,2,\ldots ,n-1$ and $0<l\le k$ . Then, for $0<j\le n$, it follows from (6.6) that

$$\begin{aligned} a_{j}^{(n,\nu )}= & {} -q^{2-\nu }\sum _{\lambda =0}^{n-j}q^{2(n-1-\lambda )}a_{0}^{(\lambda ,\nu )}(-1)^{j-1}q^{(j-1)(j-\nu )}\sum _{i=0}^{\left[ \frac{n-j-\lambda }{2}\right] }a_{0}^{(n-j-\lambda -2i,\nu )}c_{\lambda ,i} \\= & {} (-1)^{j}q^{j(j+1-\nu )}\sum _{\lambda =0}^{n-j}\left( \sum _{i=0}^{\left[ \frac{n-j-\lambda }{2}\right] }\big (q^{2}\big ) ^{n-j-\lambda }a_{0}^{(\lambda ,\nu )}a_{0}^{(n-j-\lambda -2i,\nu )}c_{\lambda ,i}\right) , \end{aligned}$$

with $$\;c_{\lambda ,i}=q^{2i}\frac{\left( \left( q^{2}\right) ^{j-1};q^{2}\right) _{i}}{\left( q^{2};q^{2}\right) _{i}}\frac{\left( \left( q^{2}\right) ^{j};q^{2}\right) _{n-j-\lambda -2i}}{\left( q^{2};q^{2}\right) _{n-j-\lambda -2i}}\frac{\left( \left( q^{2}\right) ^{n-\lambda -2i};q^{2}\right) _{i}}{\left( \left( q^{2}\right) ^{n-j-\lambda -2i+2};q^{2}\right) _{i}}$$. Hence

$$\begin{aligned} a_{j}^{(n,\nu )}=(-1)^{j}q^{j(j+1-\nu )}\sum _{i=0}^{\left[ \frac{n-j}{2} \right] }\left( \sum _{\lambda =0}^{n-j-2i}\big (q^{2}\big )^{n-j-\lambda }a_{0}^{(\lambda ,\nu )}a_{0}^{(n-j-\lambda -2i,\nu )}c_{\lambda ,i}\right) . \end{aligned}$$

Setting $\;\gamma _{\lambda ,i}=\big (q^{2}\big )^{n-j-\lambda -i}c_{\lambda ,i}\;$ in the last identity and using Lemma 3.5 yields

$$\begin{aligned} a_{j}^{(n,\nu )}=(-1)^{j}q^{j(j+1-\nu )}\sum _{\lambda =0}^{\left[ \frac{n-j}{ 2}\right] }\big (q^{2}\big )^{i}\left( \sum _{\theta =0}^{\left[ \frac{n-j}{2} \right] -i}a_{0}^{(n-j-2i-2\theta ,\nu )}\left( \sum _{\lambda =0}^{n-j-2i-\theta }\gamma _{\lambda ,i}\right) \right) , \end{aligned}$$

which can be rewritten as

$$\begin{aligned} a_{j}^{(n,\nu )}= & {} (-1)^{j}q^{j(j+1-\nu )}\sum _{i=0}^{\left[ \frac{n-j}{2}\right] }a_{0}^{(n-j-2i,\nu )}\big (q^{2}\big )^{i}\frac{\left( \big (q^{2}\big )^{j};q^{2}\right) _{i}}{\left( q^{2};q^{2}\right) _{i}}\\&\times \left( \sum _{\theta =0}^{i}\big (q^{2}\big ) ^{2\theta }\frac{\left( \big (q^{2}\big )^{j-1};q^{2}\right) _{\theta }}{ \left( q^{2};q^{2}\right) _{\theta }}\right. \\&\times \left. \left( \sum _{\lambda =0}^{n-j-2i}\!\big ( q^{2}\big )^{\lambda }\frac{\left( \big (q^{2}\big )^{j};q^{2}\right) _{i+\lambda }}{\left( q^{2};q^{2}\right) _{i+\lambda }}\frac{\left( \big ( q^{2}\big )^{1+i+\lambda -\theta };q^{2}\right) _{1}}{\left( \big (q^{2}\big ) ^{1+i+\lambda };q^{2}\right) _{1}}\right) \right) . \end{aligned}$$

Finally, we use Lemma 3.3 and the proposition follows. $\square$

#### Remark 6.3

Notice that Proposition 6.2 also holds true for every nonnegative integers *n* and *j* since, when $j>n$, then, by (6.3), both members become identically zero.

#### Lemma 6.4

For $n=0,1,2,\ldots$ and $\nu >0$ fixed, the sequence $\left\{ J_{\nu }\big (q^{1+n}j_{k\nu };q^{2}\big )\right\} _{k\in \mathbb {N}}$ is uniformly bounded (with respect to *n*).

#### Proof

One must show that there exists *C*, independent of *k* and *n*, such that

$$\begin{aligned} \left| J_{\nu }\big (q^{1+n}j_{k\nu };q^{2}\big )\right| \le C \end{aligned}$$

for every *k* and *n*. Using Theorem A we may write, for *k* large enough,

$$\begin{aligned} J_{\nu }\big (q^{1+n}j_{k\nu };q^{2}\big )=J_{\nu }\big (q^{1+n-k+\epsilon _{k}^{(\nu )}};q^{2}\big ). \end{aligned}$$

This suggests dividing the proof in two cases, according to the behavior of $n-k$ .

(i) When $k-n>0$ is sufficiently large then, by Corollary 3 of [26],

$$\begin{aligned} j_{k-n-1,\nu }=q^{1+n-k+\epsilon _{k-n-1}^{(\nu )}}<q^{1+n-k+\epsilon _{k}^{(\nu )}}=q^{1+n}j_{k\nu }<q^{1+n-k}. \end{aligned}$$

Therefore, if $n\in \mathbb {N}$ then $q^{1+n}j_{k\nu }\in \left] j_{k-n-1,\nu },\,q^{1+n-k}\right[$, whenever $k-n>0$ is sufficiently large. Now, by definition,

6.7 $$\begin{aligned} J_{\nu }\big (j_{k-n-1,\nu };q^{2}\big )=0, \end{aligned}$$

and, by virtue of (12) in [18, p. 1205], for large (positive) values of $k-n$,

6.8 $$\begin{aligned} J_{\nu }\big (q^{1+n-k};q^{2}\big )\le \frac{\left( -q^{2},-q^{2(\nu +1)};q^{2}\right) _{\infty }}{\left( q^{2};q^{2}\right) _{\infty }} q^{(k-n+\nu )(k-n-1)}. \end{aligned}$$

Moreover, it follows from Theorem A that

$$\begin{aligned} \left] j_{k-n-1,\nu },\,q^{1+n-k}\right[ \subset \left] q^{1+n-k+\alpha _{k-n-1}^{(\nu )}},q^{1+n-k}\right[ . \end{aligned}$$

By Corollary 2 of [26], the function $J_{\nu }\big (x;q^{2}\big )$ is monotone in the interval $\left] j_{k-n-1,\nu },\,q^{1+n-k}\right[$. Hence, by (6.7) and (6.8), $\left| J_{\nu }\big ( q^{1+n}j_{k\nu };q^{2}\big )\right|$ is bounded whenever $k-n$ is sufficiently large (positive).

(ii) Now, let us consider $k-n$ bounded above. Then $n-k$ is bounded below. Thus, if $\nu >0$, $\left| J_{\nu }\big ( q^{1+n}j_{k\nu };q^{2}\big )\right| =\left| J_{\nu }\big ( q^{1+n-k+\epsilon _{k}^{(\nu )}};q^{2}\big )\right|$ is bounded. $\square$

### Behavior of $J_{\nu }^{\prime }\big (qj_{k\nu };q^{2}\big )$

The asymptotic behavior of $J_{\nu }^{\prime }\big (qj_{k\nu };q^{2}\big )$ when $k\rightarrow \infty$ was recently investigated [26, Lemma 1], combining the asymptotic properties of the infinite *q*-shifted factorial $(z;q)_{\infty }$ from [29] with the ideas developed in [47]. We provide here a more direct proof, based only on the definition of the Hahn–Exton *q*-Bessel function and its derivative.

#### Lemma 6.5

For large values of *k*,

$$\begin{aligned} J_{\nu }^{\prime }(j_{k\nu };q^{2})=A_{\nu }(q)\,q^{-\big (k+\frac{\nu }{2} -1-\epsilon _{k}^{(\nu )}\big )^{2}}S_{k}, \end{aligned}$$

where $$\;A_{\nu }(q)\!=\!\frac{2\big (q^{2(\nu +1)};q^2\big )_{\infty }}{\big ( q^2;q^2\big )_{\infty }} \,q^{\frac{(\nu -1)(\nu -3)}{4}}\;$$ and $$\;\liminf _{k\rightarrow \infty }|S_k|\!>\!0.$$

#### Proof

We will present only the main steps of the proof. Computing the derivative of the function $J_{\nu }(z;q^{2})$ and setting $z=j_{k\nu }$, gives

$$\begin{aligned} J_{\nu }^{\prime }(j_{k\nu };q^{2})=\frac{2\big (q^{2(\nu +1)};q^{2}\big ) _{\infty }}{\big (q^{2};q^{2}\big )_{\infty }}\sum \limits _{n=0}^{\infty }(-1)^{n}\frac{nq^{n(n+1)}}{(q^{2(\nu +1)},q^{2};q^{2})_{n}}\big (j_{k\nu } \big )^{2n+\nu -1}. \end{aligned}$$

By Theorem A, $j_{k\nu }=q^{-k+\epsilon _{k}^{(\nu )}}$, and the above identity becomes

6.9 $$\begin{aligned} J_{\nu }^{\prime }(j_{k\nu };q^{2})=A_{\nu }(q)\,q^{-\big (k+\frac{\nu }{2} -1-\epsilon _{k}^{(\nu )}\big )^{2}}S_{k}\text {,} \end{aligned}$$

where $\;A_{\nu }(q)\!=\!\frac{2\big (q^{2(\nu +1)};q^{2}\big )_{\infty }}{ \big (q^{2};q^{2}\big )_{\infty }}\,q^{\frac{(\nu -1)(\nu -3)}{4}}\;$ and $S_{k}=\sum \limits _{n=0}^{\infty }(-1)^{n}\frac{nq^{\big ( n-k+1/2+\epsilon _{k}^{(\nu )}\big )^{2}}}{(q^{2(\nu +1)},q^{2};q^{2})_{n}}$ . Considering $m=n-k$, straightforward manipulations lead to

$$\begin{aligned} (-1)^{k}S_{k}=\sum \limits _{m=-k}^{\infty }(-1)^{m}\frac{mq^{\big ( m+1/2+\epsilon _{k}^{(\nu )}\big )^{2}}}{(q^{2(\nu +1)},q^{2};q^{2})_{m+k}}. \end{aligned}$$

Thus, if $\ p$ is a positive integer,

$$\begin{aligned} (-1)^{k}S_{k}=\sum \limits _{m=-k}^{-(2p+2)}F_{m,k}^{(\nu )}(q)+\sum \limits _{m=-(2p+1)}^{2p}F_{m,k}^{(\nu )}(q)+\sum \limits _{m=2p+1}^{\infty }F_{m,k}^{(\nu )}(q)\text {,} \end{aligned}$$

with

$$\begin{aligned} F_{m,k}^{(\nu )}(q)=(-1)^{m}\frac{mq^{\big (m+1/2+\epsilon _{k}^{(\nu )}\big ) ^{2}}}{(q^{2(\nu +1)},q^{2};q^{2})_{m+k}}. \end{aligned}$$

Hence,

$$\begin{aligned} \left| S_{k}\right| \ge \left| \sum \limits _{m=-(2p+1)}^{2p}F_{m,k}^{(\nu )}(q)\right| -\sum \limits _{m=-k}^{-(2p+2)}\left| F_{m,k}^{(\nu )}(q)\right| -\sum \limits _{m=2p+1}^{\infty }\left| F_{m,k}^{(\nu )}(q)\right| . \end{aligned}$$

Since this estimate is independent of *p*, one can resort to (2.6)–(2.8) to show that the last two sums on the right side of the previous inequality tend to zero when $k\rightarrow \infty$. Moreover, as $k\rightarrow \infty$,

$$\begin{aligned} \begin{array}{lll} \sum \limits _{m=-(2p+1)}^{2p}F_{m,k}^{(\nu )}(q) &{} = &{} \frac{1}{\big (q^{2(\nu +1)},q^{2};q^{2}\big )_{\infty }}\sum \limits _{m=-(2p+1)}^{2p}(-1)^{m}mq^{\big ( m+1/2\big )^{2}}+o(1) \\ &{} = &{} \frac{q^{1/4}}{\big (q^{2(\nu +1)},q^{2};q^{2}\big )_{\infty }} \sum \limits _{m=0}^{2p}(-1)^{m}(2m+1)q^{m(m+1)}+o(1). \end{array} \end{aligned}$$

Therefore,

6.10 $$\begin{aligned} \liminf _{k\rightarrow \infty } \left| S_{k}\right| \ge \frac{q^{1/4}}{\big (q^{2(\nu +1)},q^{2};q^{2}\big )_{\infty }}\, \left| \sum \limits _{i=0}^{\infty }(-1)^{i}(2i+1)q^{i(i+1)}\right| . \end{aligned}$$

Identity (10.4.9) of Corollary 10.4.2 due to Jacobi [15, p. 500] guarantees that

6.11 $$\begin{aligned} \sum \limits _{i=0}^{\infty }(-1)^{i}(2i+1)q^{i(i+1)}=\prod _{i=1}^{\infty } \big (1-q^{2i}\big )^{3}>0. \end{aligned}$$

The lemma now follows from (6.9), (6.10) and (6.11). $\square$

Notice that the above lemma implies that $J_{\nu }^{\prime }(j_{k\nu };q^{2})=\mathcal {O}\left( q^{-k(k+\nu -2)}\right)$ as $k\rightarrow \infty$.

### Sufficient conditions

Recall the notation $V_{q}^{+}\,=\,\left\{ q^{n}:\,n=0,1,2,\ldots \right\}$ for the support points of the *q*-integral (2.3) in [0, 1] and the concept of a *q*-linear Hölder function in $V_{q}^{+}$ defined in the introduction. In [25] the following upper bound for basic Fourier–Bessel coefficient (4.2) has been obtained. However, the uniform convergence of basic Fourier–Bessel expansions requires the slightly more restrictive conditions of Theorem 1.2 .

#### Theorem 6.6

If the function f is *q*-linear Hölder of order $\lambda >0$ in $V_{q}^{+}\cup \{q^{-1}\},$ with $t^{-\frac{1}{2}}f(t)\in L_{q}^{2}(0,1)$ and the limit $\lim _{x\rightarrow 0^{+}}f(x)=f(0^{+})$ is finite, then

$$\begin{aligned}&\left| \int _{0}^{1}tf(t)J_{\nu }(qj_{k\nu }t;q^{2})d_{q}t\right| \\&\quad \le \frac{(1-q)q^{\nu -1}}{j_{k\nu }}\left| f \big (q^{-1}\big )J_{\nu +1}(qj_{k\nu };q^{2})\right| \\&\qquad +\frac{(1-q)q^{\frac{\nu -3}{2}}}{j_{k\nu }}\eta _{k,\nu }^{\frac{1}{2}}\left( \frac{q^{\frac{\nu +1}{2}}M_{1}}{(1-q)^{\frac{1 }{2}}\big (1-q^{2\alpha }\big )^{\frac{1}{2}}}+\frac{q^{\frac{\nu }{2}}-q^{- \frac{\nu }{2}}}{q^{\frac{1}{2}}-q^{-\frac{1}{2}}}\sqrt{M_{2}}\right) , \end{aligned}$$

where $M_{1}$ and $M_{2}$ are independent of *k* and $\eta _{k,\nu }$ is given by (4.3).

The proof of the main result depends on a refinement of the above estimates. We start proving the following identity.

#### Lemma 6.7

Let $\nu >0$. If *f* is a function such that $\lim _{x\rightarrow 0^{+}}f(x)<+\infty$, then

$$\begin{aligned}&\int _{0}^{1}tf(t)J_{\nu }(qj_{k\nu }t;q^{2})d_{q}t\\&\quad =\frac{ (1-q)q^{\nu -2}f\big (q^{-1}\big )J_{\nu }(qj_{k\nu };q^{2})}{j_{k\nu }^{2}} \\&\qquad - \, \frac{q^{\nu -2}}{j_{k\nu }^{2}}\left[ \left( q^{ \frac{\nu }{2}}-q^{-\frac{\nu }{2}}\right) \left( q^{\frac{\nu }{2} }\int _{0}^{1}J_{\nu }(qj_{k\nu }t;q^{2})\frac{f(qt)}{t}d_{q}t\right. \right. \\&\qquad \left. -\,q^{-\frac{\nu }{2}}\int _{0}^{1}J_{\nu }(qj_{k\nu }t;q^{2})\frac{f(t)}{t}d_{q}t\right) \\&\qquad - \, q^{\frac{\nu }{2}}\left( q^{\frac{\nu }{2} }\int _{0}^{1}J_{\nu }(qj_{k\nu }t;q^{2})\frac{f(qt)-f(t)}{t}d_{q}t\right. \\&\qquad \left. \left. - \, q^{-\frac{ \nu }{2}}\int _{0}^{1}J_{\nu }(qj_{k\nu }t;q^{2})\frac{f(t)-f(t/q)}{t} d_{q}t\right) \right] , \end{aligned}$$

provided all *q*-integrals converge.

#### Proof

We will use the symmetric operator $\delta _{q}$ notation (2.1). From (3.7) in [25, Proposition 4, p. 329],

$$\begin{aligned} \frac{\delta _{q}\left[ x^{\nu }J_{\nu }\big (x;q^{2}\big )\right] }{\delta _{q}x}=\frac{q^{-\frac{\nu }{2}}}{1-q}x^{\nu }J_{\nu -1}\big (q^{-\frac{1}{2} }x;q^{2}\big ), \end{aligned}$$

and, using *q*-integration by parts (2.4) together with the assumption $\lim _{x\rightarrow 0^{+}}f(x)=f(0^{+})<+\infty$,

6.12 $$\begin{aligned}&\int _{0}^{1}tf(t)J_{\nu }(qj_{k\nu }t;q^{2})d_{q}t\nonumber \\&\quad =\frac{ (1-q)q^{\nu -1}f\big (q^{-1}\big )J_{\nu +1}(qj_{k\nu };q^{2})}{j_{k\nu }}- \frac{(1-q)q^{\frac{\nu -3}{2}}}{j_{k\nu }} \nonumber \\&\qquad \times \left[ -\frac{q^{\frac{\nu }{2}}-q^{-\frac{\nu }{2}} }{q^{\frac{1}{2}}-q^{-\frac{1}{2}}}\int _{0}^{1}\!J_{\nu +1}(qj_{k\nu }t;q^{2})f(t)d_{q}t+q^{\frac{\nu }{2}}\!\int _{0}^{1}\!J_{\nu +1}(qj_{k\nu }t;q^{2})f_{2}(t)d_{q}t\right] ,\nonumber \\ \end{aligned}$$

where

6.13 $$\begin{aligned} f_{2}(t):=\frac{t\delta _{q}f\big (q^{-\frac{1}{2}}t\big )}{\delta _{q}t}, \end{aligned}$$

and the operator $\delta _{q}$ is given in (2.1). We will now rewrite the integrals on the right hand side of (6.12). Using (3.8) of [25, Proposition 4, p. 329], (2.4) and $\lim _{x\rightarrow 0^{+}}f(x)=f(0^{+})$, the first integral on the right hand side of (6.12) becomes

$$\begin{aligned} \int _{0}^{1}\!J_{\nu +1}(qj_{k\nu }t;q^{2})f(t)d_{q}t=\frac{(1-q)q^{\frac{ \nu -3}{2}}}{j_{k\nu }}\int _{0}^{1}\!J_{\nu }(qj_{k\nu }t;q^{2})\frac{ t^{-\nu }\delta _{q}\left[ t^{\nu }f\big (q^{\frac{1}{2}}t\big )\right] }{ \delta _{q}t}d_{q}t. \end{aligned}$$

Evaluating the right hand side using (2.2 ),

6.14 $$\begin{aligned} \int _{0}^{1}\!J_{\nu +1}(qj_{k\nu }t;q^{2})f(t)d_{q}t= & {} -\frac{q^{ \frac{\nu -2}{2}}}{j_{k\nu }}\left[ q^{\frac{\nu }{2}}\int _{0}^{1}\!J_{\nu }(qj_{k\nu }t;q^{2})\frac{f(qt)}{t}d_{q}t\right. \nonumber \\&\left. - \, q^{-\frac{\nu }{2} }\int _{0}^{1}\!J_{\nu }(qj_{k\nu }t;q^{2})\frac{f(t)}{t}d_{q}t\right] . \end{aligned}$$

The evaluation of the second *q*-integral on the right side of (6.12) is similar and provides

6.15 $$\begin{aligned}&\int _{0}^{1}\!J_{\nu +1}(qj_{k\nu }t;q^{2})f_{2}(t)d_{q}t\nonumber \\&\quad =\frac{ q^{\frac{\nu -1}{2}}}{\left( 1-q\right) j_{k\nu }} \nonumber \\&\qquad \times \left[ q^{\frac{\nu }{2}}\int _{0}^{1}\!J_{\nu }(qj_{k\nu }t;q^{2})\frac{f(qt)-f(t)}{t}d_{q}t\right. \nonumber \\&\qquad \left. - \, q^{-\frac{\nu }{2} }\int _{0}^{1}\!J_{\nu }(qj_{k\nu }t;q^{2})\frac{f(t)-f\big (\frac{t}{q}\big )}{ t}d_{q}t\right] . \end{aligned}$$

Finally, the lemma follows by substituting (6.14) and (6.15) into (6.12) and using the identity (4.4). $\square$

#### Remark 6.8

To assure the convergence of all *q*-integrals involved in the previous lemma, one is required to assume the same sufficient conditions of Theorem 1.2.

We are now in conditions to prove our main result. The beef of the proof consists of finding an *n*-independent (convergent) upper bound for the *q*-Fourier–Bessel series.

#### Proof of Theorem 1.2

As observed before, the assumptions of Theorem 1.2 allow the use of Lemma 6.7, yielding

6.16 $$\begin{aligned}&\left| \int _{0}^{1}tf(t)J_{\nu }(qj_{k\nu }t;q^{2})d_{q}t\right| \nonumber \\&\quad \le \frac{(1-q)q^{\nu -2}\left| f\big (\frac{1 }{q}\big )J_{\nu }(qj_{k\nu };q^{2})\right| }{j_{k\nu }^{2}} \nonumber \\&\qquad +\, \frac{q^{\nu -2}}{j_{k\nu }^{2}}\left[ \left| q^{\frac{\nu }{2}}-q^{-\frac{\nu }{2}}\right| \left( q^{\frac{\nu }{2} }\left| \int _{0}^{1}J_{\nu }(qj_{k\nu }t;q^{2})\frac{f(qt)}{t} d_{q}t\right| \right. \right. \nonumber \\&\qquad \left. \left. +\,q^{-\frac{\nu }{2}}\!\left| \int _{0}^{1}J_{\nu }(qj_{k\nu }t;q^{2})\frac{f(t)}{t}d_{q}t\right| \right) \right. \nonumber \\&\qquad +\,\left. q^{\frac{\nu }{2}}\left( q^{\frac{\nu }{2} }\left| \int _{0}^{1}J_{\nu }(qj_{k\nu }t;q^{2})\frac{f(qt)-f(t)}{t} d_{q}t\right| \right. \right. \nonumber \\&\qquad \left. \left. +\,q^{-\frac{\nu }{2}}\left| \int _{0}^{1}J_{\nu }(qj_{k\nu }t;q^{2})\frac{f(t)-f\big (\frac{t}{q}\big )}{t}d_{q}t\right| \right) \right] \text {.} \end{aligned}$$

Employing the *q*-Hölder type inequality [27, Th. 3.4, p. 346] with $p=2$, the four *q*-integrals appearing on the right side of (6.16) can be estimated as follows:

6.17 $$\begin{aligned} \left| \int _{0}^{1}J_{\nu }(qj_{k\nu }t;q^{2})\frac{f(qt)}{t }d_{q}t\right|\le & {} \left( \int _{0}^{1}tJ_{\nu }^{2}(qj_{k\nu }t;q^{2})d_{q}t\right) ^{\frac{1}{2}}\nonumber \\ \left( \int _{0}^{1}\frac{f^{2}(qt)}{t^{3}}d_{q}t\right) ^{\frac{1}{2}}\le & {} \eta _{k\nu }^{\frac{1}{2}}\left( \int _{0}^{1}\frac{ f^{2}(qt)}{t^{3}}d_{q}t\right) ^{\frac{1}{2}}\text {,} \end{aligned}$$

6.18 $$\begin{aligned} \left| \int _{0}^{1}J_{\nu }(qj_{k\nu }t;q^{2})\frac{f(t)}{t} d_{q}t\right|\le & {} \eta _{k\nu }^{\frac{1}{2}}\left( \int _{0}^{1}\frac{f^{2}(t)}{t^{3}}d_{q}t\right) ^{\frac{1}{2}}, \end{aligned}$$

6.19 $$\begin{aligned} \left| \int _{0}^{1}J_{\nu }(qj_{k\nu }t;q^{2})\frac{f(qt)-f(t)}{t} d_{q}t\right|\le & {} \eta _{k\nu }^{\frac{1}{2}}\left( \int _{0}^{1}\frac{\left( f(qt)-f(t)\right) ^{2}}{t^{3}}d_{q}t\right) ^{\frac{ 1}{2}}, \nonumber \\ \end{aligned}$$

6.20 $$\begin{aligned} \left| \int _{0}^{1}J_{\nu }(qj_{k\nu }t;q^{2})\frac{f(t)-f\big (\frac{t}{q }\big )}{t}d_{q}t\right|\le & {} \eta _{k\nu }^{\frac{1}{2} }\left( \int _{0}^{1}\frac{\left( f(t)-f\big (\frac{t}{q}\big )\right) ^{2}}{ t^{3}}d_{q}t\right) ^{\frac{1}{2}}. \nonumber \\ \end{aligned}$$

Now, by virtue of the assumption $t^{-\frac{3}{2}}f(t)\in L_{q}^{2}[0,1]$, one can write the *q*-integral as an infinite convergent sum

6.21 $$\begin{aligned}&\int _{0}^{1}\frac{f^{2}(qt)}{t^{3}}d_{q}t\nonumber \\&\quad =(1-q)\sum _{n=0}^{\infty }\frac{ q^{n}f^{2}(q^{n+1})}{q^{3n}}\nonumber \\&\quad =(1-q)q^{2}\sum _{n=0}^{\infty }\left( \frac{ f(q^{n+1})}{q^{n+1}}\right) ^{2}=S<+\infty \text {,} \end{aligned}$$

and

6.22 $$\begin{aligned} \int _{0}^{1}\frac{f^{2}(t)}{t^{3}}d_{q}t=(1-q)\sum _{n=0}^{\infty }\left( \frac{f(q^{n})}{q^{n}}\right) ^{2}=T<+\infty \,\text {.} \end{aligned}$$

Moreover, since *f* is *q*-linear Hölder of order $\lambda >1$ in $V_{q}^{+}\cup \left\{ q^{-1}\right\}$, there exist constants *U* and *W* such that

6.23 $$\begin{aligned} \int _{0}^{1}\frac{\left( f(qt)-f(t)\right) ^{2}}{t^{3}}d_{q}t= & {} (1-q)\sum _{n=0}^{\infty }\frac{\Big (f\big (q^{n+1}\big )-f \big (q^{n}\big )\Big )^{2}}{q^{2n}} \nonumber \\\le & {} (1-q)U^{2}\sum _{n=0}^{\infty }\frac{q^{2\alpha n}}{ q^{2n}}=\frac{(1-q)U^{2}}{1-q^{2(\alpha -1)}}, \end{aligned}$$

and

6.24 $$\begin{aligned} \int _{0}^{1}\frac{\left( f(t)-f\big (\frac{t}{q}\big )\right) ^{2} }{t^{3}}d_{q}t= & {} (1-q)\sum _{n=0}^{\infty }\frac{\Big (f\big ( q^{n}\big )-f\big (q^{n-1}\big )\Big )^{2}}{q^{2n}} \nonumber \\\le & {} (1-q)W^{2}\sum _{n=0}^{\infty }\frac{q^{2\alpha n}}{ q^{2n}}=\frac{(1-q)W^{2}}{1-q^{2(\alpha -1)}}. \end{aligned}$$

The constants $S\equiv S_{q}(f)$, $T\equiv T_{q}(f)$, $U\equiv U_{q}(f)$ and $W\equiv W_{q}(f)$ in (6.21), (6.21), (6.23) and (6.24) are independent of *k*. Notice also that the extra condition involving the point $q^{-1}$ can be removed (or neglected) since it only affects the choice of the constant *W*.

Introducing inequalities (6.21), (6.22), (6.23) and (6.24) into inequalities (6.17), (6.18), (6.19) and (6.20), respectively, and using (6.16), gives:

$$\begin{aligned} \left| \int _{0}^{1}tf(t)J_{\nu }(qj_{k\nu }t;q^{2})d_{q}t\right|\le & {} \frac{(1-q)q^{\nu -2}\left| f\big (\frac{1 }{q}\big )J_{\nu }(qj_{k\nu };q^{2})\right| }{j_{k\nu }^{2}} \\&+ \, \frac{q^{\nu -2}\eta _{k}^{\frac{1}{2}}}{j_{k\nu }^{2}}\left[ \left| q^{\frac{\nu }{2}}-q^{-\frac{\nu }{2}}\right| \left( q^{\frac{ \nu }{2}}\sqrt{S}+q^{-\frac{\nu }{2}}\sqrt{T}\right) \right. \\&\left. + \,q^{\frac{ \nu }{2}}\left( q^{\frac{\nu }{2}}\sqrt{\frac{(1-q)U^{2}}{1-q^{2(\alpha -1)}} }+q^{-\frac{\nu }{2}}\sqrt{\frac{(1-q)W^{2}}{1-q^{2(\alpha -1)}}}\right) \right] , \end{aligned}$$

or, equivalently,

$$\begin{aligned} \left| \int _{0}^{1}tf(t)J_{\nu }\big (qj_{k\nu }t;q^{2}\big ) d_{q}t\right| \le \frac{1}{j_{k\nu }^{2}}\left\{ C_{1}\left| J_{\nu }\big (qj_{k\nu };q^{2}\big )\right| +C_{2}\left( \eta _{k,\nu }\right) ^{ \frac{1}{2}}\right\} , \end{aligned}$$

where $C_{1}$ and $C_{2}$ depend on *f*, $\nu$ and *q*, but not on *k*. Thus, the absolute value of the $k^{th}$ term in (6.1) can be bounded as follows:

6.25 $$\begin{aligned}&\left| a_{k}^{(\nu )}\left( f\right) J_{\nu }\big (q^{n+1}j_{k\nu };q^{2} \big )\right| \nonumber \\&\quad =\left| \left( \frac{1}{\eta _{k,\nu }} \int _{0}^{1}t f(t)J_{\nu }\big (qj_{k\nu }t;q^{2}\big )d_{q}t\right) J_{\nu } \big (q^{n+1}j_{k\nu };q^{2}\big )\right| \nonumber \\&\quad \le \left\{ \frac{C_{1}\left| J_{\nu }\big (qj_{k\nu };q^{2}\big ) \right| }{j_{k\nu }^{2}\eta _{k,\nu }}+\frac{C_{2}}{j_{k\nu }^{2}\eta _{k,\nu }^{\frac{1}{2}}}\right\} \left| J_{\nu }\big (q^{1+n}j_{k\nu };q^{2}\big )\right| \nonumber \\&\quad \le \left\{ \frac{qC_{1}}{j_{k\nu }\left| J_{\nu }^{\prime }\big ( j_{k\nu };q^{2}\big )\right| }+\frac{q^{\frac{1}{2}}C_{2}}{j_{k\nu }^{ \frac{3}{2}}\left| J_{\nu }^{\prime }\big (j_{k\nu };q^{2}\big ) \right| ^{\frac{1}{2}}\left| J_{\nu }\big (qj_{k\nu };q^{2}\big ) \right| ^{\frac{1}{2}}}\right\} \left| J_{\nu }\big (q^{1+n}j_{k\nu };q^{2}\big )\right| \text {,}\nonumber \\ \end{aligned}$$

using (4.3). To deal with the first term in (6.25), one uses Theorem A and Lemmas 6.4 and 6.5 to assure the existence of a constant $M>0$, independent of *k* and *n*, such that

$$\begin{aligned} \frac{\left| J_{\nu }\big (q^{1+n}j_{k\nu };q^{2}\big )\right| }{ j_{k\nu }\left| J_{\nu }^{\prime }\big (j_{k\nu };q^{2}\big )\right| } \le Mq^{k}. \end{aligned}$$

By Proposition 6.1, the second term in (6.25) reads

$$\begin{aligned} \frac{\left| J_{\nu }\big (q^{1+n}j_{k\nu };q^{2}\big )\right| }{ j_{k\nu }^{\frac{3}{2}}\left| J_{\nu }^{\prime }\big (j_{k\nu };q^{2}\big ) \right| ^{\frac{1}{2}}\left| J_{\nu }\big (qj_{k\nu };q^{2}\big ) \right| ^{\frac{1}{2}}}=\frac{\left| J_{\nu }\big (qj_{k\nu };q^{2} \big )\right| ^{\frac{1}{2}}}{j_{k\nu }^{\frac{3}{2}}\left| J_{\nu }^{\prime }\big (j_{k\nu };q^{2}\big )\right| ^{\frac{1}{2}}}\left| P_{n}\big (j_{k\nu }^{2};q\big )\right| . \end{aligned}$$

From (6.2), Proposition  and (6.4) it follows that, for all *n*,

$$\begin{aligned} \lim _{k\rightarrow \infty }\left| \frac{P_{n}\big (j_{k\nu }^{2};q\big )}{ a_{n}^{(n,\nu )}(j_{k\nu }^{2})^{n}}\right| =1,\quad \hbox {and }\quad P_{n}\big (j_{k\nu }^{2};q\big )=\mathcal {O}\left( q^{n(n+1-\nu )}j_{k\nu }^{2n}\right) \quad \hbox {as}\quad k\rightarrow \infty . \end{aligned}$$

Combining Theorems A and C with Lemma 6.5, this allows to bound the second term in (6.25),

$$\begin{aligned} Aq^{k^{2}+\nu k+n(n+1-\nu )-2kn}=Aq^{\left( n-k-\frac{\nu -1}{2}\right) ^{2}- \frac{(\nu -1)^{2}}{4}}q^{k}\le Bq^{k}, \end{aligned}$$

since $\epsilon _{k}^{(\nu )}(q)>0$ and $\epsilon _{k}^{(\nu )}(q)=\mathcal {O }(q^{2k})$ when $k\rightarrow \infty$ (see (2.8) and (2.6)). Since the constants *A* and *B* are positive and independent of *k* and *n*, we finally conclude that

$$\begin{aligned} \left| a_{k}^{(\nu )}\left( f\right) J_{\nu }\big (q^{n+1}j_{k\nu };q^{2} \big )\right| =\mathcal {O}\left( q^{{k}}\right) \quad \hbox {as}\quad k\rightarrow \infty \text {.} \end{aligned}$$

This proves the uniform convergence of the basic Fourier–Bessel series (4.1) on the set $V_{q}^{+}$. $\square$

#### Remark 6.9

The $\ q$-linear Hölder condition can be slightly relaxed. Indeed, it suffices that *f* satisfies the *almostq*-linear Hölder condition [24, p. 105], which means that the condition only needs to be satisfied for all integers *n* such that $n\ge n_{0}$, where $n_{0}$ is a positive integer.

## Examples

We conclude with some explicit examples of uniformly convergent Fourier–Bessel series on a *q*-linear grid.

### Example 7.1

Consider $f(x):=x^{\nu }$. Using the power series expansion of $J_{\nu }(x;q^{2})$ and the definition of the *q*-integral, a calculation shows that

$$\begin{aligned} \int _{0}^{1}t^{\nu +1}J_{\nu }(qj_{k\nu }t;q^{2})d_{q}t=\frac{1-q}{qj_{k\nu } }J_{\nu +1}(qj_{k\nu };q^{2}) \end{aligned}$$

and (4.2)–(4.3) gives

$$\begin{aligned} a_{n}(x^{\nu })=-\frac{2}{q^{\nu }j_{k\nu }}\frac{1}{J_{\nu }^{\prime }(j_{k\nu };q^{2})}. \end{aligned}$$

It is straightforward to check that the function $f(x)=x^{\nu }$ is *q* -linear Hölder of order $\nu$ and, if $\nu >1$, that

$$\begin{aligned} x^{-\frac{3}{2}}f(x)=x^{\nu -\frac{3}{2}}\in L_{q}^{2}[0,1],\qquad \lim _{x\rightarrow 0^{+}}x^{\nu }=0. \end{aligned}$$

Thus, by Theorem 1.2, we conclude that the *q* -Fourier–Bessel series $S_{q}^{(\nu )}\big [x^{\nu }\big ]$ converges uniformly on $\,V_{q}^{+}=\left\{ q^{n}:\,n=0,1,2,\ldots \,\right\}$ whenever $\nu >1$. Hence, by Theorem 5.2, we have

$$\begin{aligned} x^{\nu }=-2\,q^{-\nu }\sum _{k=1}^{\infty }\frac{J_{\nu }(qj_{k\nu }x;q^{2})}{ j_{k\nu }J_{\nu }^{\prime }(j_{k\nu };q^{2})},\qquad x=q^{n},\,\,n=0,1,2,\ldots . \end{aligned}$$

The convergence of the expansion of $x^{\nu }$ in classical Fourier–Bessel series was studied in [48, §18.22] using contour integral methods.

### Example 7.2

Consider $g_{\nu ,\mu }(x;q)\equiv g(x;q)\!:=\!x^{\nu }\frac{ (x^{2}q^{2};q^{2})_{\infty }}{(x^{2}q^{2\mu -2\nu };q^{2})_{\infty }}$, with $|x|<1$ and $\mu>\nu >-\frac{1}{2}$. Using the *q*-binomial Theorem [31, (1.3.2)] we have

$$\begin{aligned} g(x;q)=\frac{x^{\nu }}{1-x^{2}}\left[ \sum _{n=0}^{\infty }\frac{(q^{2\mu -2\nu };q^{2})_{n}}{(q^{2};q^{2})_{n}}\,x^{2n}\right] ^{-1}. \end{aligned}$$

The limit relation

$$\begin{aligned} \lim _{q\rightarrow 1^{-}}\sum _{n=0}^{\infty }\frac{(q^{2\mu -2\nu };q^{2})_{n}}{(q^{2};q^{2})_{n}}\,x^{2n}=\sum _{n=0}^{\infty }\frac{(2\mu -2\nu )_{n}}{n!}\,x^{2n}=(1-x^{2})^{-2\mu +2\nu }\text {,} \end{aligned}$$

shows that *g*(*x*; *q*) is a *q*-analogue of $g(x)=x^{\nu }(1-x^{2})^{2\mu -2\nu -1}$. We can expand *g*(*x*; *q*) in uniform convergent *q*-Fourier–Bessel series. Setting $x=qj_{k\nu }$ in formula (4.11) from [1], and using (4.2), we find

$$\begin{aligned} \int _{0}^{1}t\,g(t;q)J_{\nu }(qj_{k\nu }t;q^{2})d_{q}t=(1-q)(qj_{k\nu })^{\nu -\mu }\frac{(q^{2};q^{2})_{\infty }}{(q^{2\mu -2\nu };q^{2})_{\infty }}J_{\mu }(qj_{k\nu };q^{2}). \end{aligned}$$

Therefore, (4.2)–(4.3) enables one to write

$$\begin{aligned} a_{k}^{(\nu )}\big (g(x;q)\big )=-2q^{1-\mu }\big (j_{k\nu }\big )^{\nu -\mu } \frac{(q^{2};q^{2})_{\infty }}{(q^{2\mu -2\nu };q^{2})_{\infty }}\frac{ J_{\mu }\big (qj_{k\nu };q^{2}\big )}{J_{\nu +1}\big (qj_{k\nu };q^{2}\big ) J_{\nu }^{\prime }\big (j_{k\nu };q^{2}\big )}. \end{aligned}$$

It can be checked that *g*(*x*; *q*) is *q*-linear Hölder of order $\nu +2$ . Also, $x^{-\frac{3}{2}}g(x;q)\in L_{q}^{2}[0,1]$ if $\nu >1$ and $\lim _{x\rightarrow 0^{+}}g(x;q)=0$. Thus, we can apply Theorem 1.2 to conclude that the *q*-Fourier series $S_{q}^{(\nu )}\big [g(x;q)\big ]$ converges uniformly on $\,V_{q}^{+}=\left\{ q^{n}:\,n=0,1,2,\ldots \,\right\}$ whenever $\nu >1$. Hence, by Theorem 5.2, we have

$$\begin{aligned} \frac{x^{\nu }(x^{2}q^{2};q^{2})_{\infty }}{(x^{2}q^{2(\mu -\nu )};q^{2})_{\infty }}= & {} -\frac{2\,q^{1-\mu }\big (q^{2};q^{2}\big )_{\infty }}{ \big (q^{2(\mu -\nu )};q^{2}\big )_{\infty }}\sum _{k=1}^{\infty }\frac{ (j_{k\nu })^{\nu -\mu }J_{\mu }\big (qj_{k\nu };q^{2}\big )J_{\nu }\big ( qj_{k\nu }x;q^{2}\big )}{J_{\nu +1}\big (qj_{k\nu };q^{2}\big )J_{\nu }^{\prime }(j_{k\nu };q^{2})},\\&\quad x=q^{n},\,\,n=0,1,2,\ldots . \end{aligned}$$

Notice that choosing $\mu =\nu +1$ in the latter example one obtains the first one.
